# NLRP3 inflammasome in cardiovascular diseases: molecular mechanisms and advances in targeted drug research

**DOI:** 10.3389/fimmu.2026.1848762

**Published:** 2026-08-04

**Authors:** Tianqing Zhang, Li Luo, Kailin Yang, Honglan Liu, Sijie Xiao, Mengmeng Wang, Junpeng Chen, Yu Zhou, Liangqing Ge

**Affiliations:** 1 Department of Cardiology, Changde Hospital, Xiangya School of Medicine, Central South University (The First People’s Hospital of Changde city), Changde, China; 2 Institute of Basic Research in Traditional Chinese Medicine, Hunan Academy of Chinese Medicine, Changsha, Hunan, China; 3 Changde Hospital Affiliated to Hengyang Medical College, University of South China, Changde, Hunan, China; 4 Department of Ultrasound, The First People’s Hospital of Changde City, Changde, China; 5 Center for Cardiometabolic Science, University of Louisville, Louisville, KY, United States

**Keywords:** cardiovascular diseases, clinical translation, immunometabolism, inflammasome inhibitors, NLRP3 inflammasome

## Abstract

The NLRP3 inflammasome plays a pivotal role in innate immune responses and has emerged as an important contributor to the pathogenesis of cardiovascular diseases (CVDs), including atherosclerosis, heart failure, myocardial infarction, and hypertension-related organ damage. Upon activation, NLRP3 promotes the maturation and release of IL-1β and IL-18 and triggers pyroptosis, thereby exacerbating endothelial dysfunction, vascular smooth muscle cell proliferation and migration, lipid metabolic disorders, and adverse myocardial remodeling. These processes form a self-perpetuating cycle of inflammation and tissue injury. Therapeutic strategies targeting the NLRP3 pathway have progressed from non-specific anti-inflammatory agents, such as aspirin and colchicine, to more precise interventions that block NLRP3 oligomerization (e.g., MCC950 and its analogs), neutralize upstream cytokines, modulate metabolic and oxidative stress pathways, and employ advanced delivery systems including gene editing and nanocarriers. Despite these advances, current approaches are limited by insufficient target specificity, incomplete long-term safety data, and the absence of disease subtype-specific regimens. This review provides a comprehensive overview of multi-level therapeutic strategies directed against the NLRP3 inflammasome, critically evaluates preclinical and clinical evidence—including findings from the CANTOS trial—and discusses the translational potential of NLRP3-targeted immunometabolic therapy in CVDs. Looking forward, integration of multi-omics technologies, such as single-cell sequencing and spatial transcriptomics, with well-designed clinical studies will be essential to delineate the context-specific roles of NLRP3 across different CVD subtypes and to clarify its interactions with protective pathways such as AMPK and SIRT1. These efforts may facilitate the development of more selective, tissue-targeted, and safer therapeutic agents for the precision management of cardiovascular diseases.

## 1 Introduction

Cardiovascular diseases (CVDs) have become the leading cause of mortality and disability worldwide. According to the World Health Organization, deaths caused by CVDs account for 31% of the global total annual deaths, and the disease burden of CVDs continues to increase with the aging of the population and the rising prevalence of metabolic syndrome (e.g., obesity, diabetes) (1). Despite significant improvements in the prognosis of CVD patients over the past few decades through traditional intervention strategies targeting dyslipidemia (e.g., statins), hypertension (e.g., angiotensin-converting enzyme inhibitors (ACEIs) and angiotensin receptor blockers (ARBs)), and thrombosis (e.g., antiplatelet drugs), nearly 40% of patients still face the risk of recurrent cardiovascular events after receiving standard treatment (2, 3). This clinical dilemma indicates that, in addition to traditional risk factors, chronic low-grade inflammation—as a core regulatory link in CVD pathogenesis—has mechanisms and therapeutic potential that have not been fully elucidated.

The innate immune response is the primary line of defense for the body against external stimuli and endogenous damage. As a key signaling complex in the innate immune pathway, the inflammasome plays a pivotal role in the process of danger signal recognition and inflammatory response initiation (4). Among them, the NLRP3 (NOD-like receptor pyrin domain-containing 3) inflammasome has become a research hotspot in the field of CVDs in recent years due to its broad range of activating stimuli (e.g., uric acid crystals, cholesterol crystals, mitochondrial damage products) and its role in various chronic inflammatory diseases (5, 6). Unlike the specific activation modes of other inflammasomes (e.g., AIM2, NLRC4), NLRP3 can be activated by a variety of endogenous damage-associated molecular patterns (DAMPs) in the pathological microenvironment of CVDs, including cholesterol crystals in atherosclerotic plaques, mitochondrial DNA (mtDNA) released during myocardial ischemia-reperfusion injury, and reactive oxygen species (ROS) released by vascular endothelial cells under hypertensive conditions (7, 8). This multi-signal response characteristic enables it to participate in multiple key pathological stages, such as fatty streak formation and plaque rupture in atherosclerosis, myocardial remodeling in heart failure, and the inflammatory phase of repair in myocardial infarction, making it a common molecular target that connects different subtypes of CVDs (9, 10).

Endothelial dysfunction is recognized as one of the earliest events in the pathogenesis of atherosclerosis and other cardiovascular diseases. Recent studies have demonstrated that NLRP3 inflammasome activation in vascular endothelial cells significantly contributes to this process. In endothelial cells, NLRP3 activation promotes the generation of reactive oxygen species, impairs nitric oxide bioavailability, upregulates adhesion molecules, and induces endothelial pyroptosis, thereby facilitating leukocyte adhesion, vascular inflammation, and plaque initiation. A comprehensive review by Bai et al. has summarized the molecular mechanisms by which the NLRP3 inflammasome drives endothelial dysfunction (11). Subsequent studies have further supported the involvement of NLRP3-mediated endothelial injury in various cardiovascular conditions, including hypertension and metabolic disorders (12–14).

Subsequent clinical studies have further confirmed that NLRP3 expression in peripheral blood monocytes of patients with CVDs positively correlates with disease severity (e.g., degree of coronary artery stenosis and cardiac function classification), and that serum levels of its downstream effector, interleukin-1β (IL-1β), independently predict recurrent cardiovascular events (15). In animal models of ischemia-reperfusion injury, inhibition of NLRP3 inflammasome assembly attenuates tissue damage (16). Collectively, these observations establish a solid rationale for targeting the NLRP3 inflammasome in CVD prevention and treatment. The landmark CANTOS trial (Canakinumab Anti-Inflammatory Thrombosis Outcomes Study), published in 2017, demonstrated that canakinumab, a monoclonal antibody against IL-1β, significantly reduced major adverse cardiovascular events (MACE) in patients with atherosclerotic cardiovascular disease (ASCVD) without affecting lipid levels (17). This trial not only verified the causal role of inflammation in CVDs but also accelerated the translation of NLRP3-targeted therapies into clinical development.

However, several important scientific questions remain unresolved. At the molecular level, NLRP3 inflammasome activation and regulation show marked heterogeneity across CVD subtypes. In atherosclerosis, activation largely depends on lysosomal rupture triggered by cholesterol crystals, whereas in heart failure it is driven primarily by mitochondrial dysfunction and mitochondrial reactive oxygen species (mtROS) release; the molecular basis for these subtype-specific patterns has not been fully elucidated (18). At the level of signal integration, crosstalk between NLRP3 and metabolic pathways such as the AMP-activated protein kinase (AMPK)/mammalian target of rapamycin (mTOR) pathway, as well as regulated cell death pathways including pyroptosis and apoptosis, remains incompletely characterized, although these interactions likely influence pathological outcomes in CVDs (18). From a drug-development perspective, existing NLRP3-targeted strategies still encounter challenges of limited target engagement (e.g., hepatic accumulation of the small-molecule inhibitor MCC950), insufficient adaptability across disease subtypes (single-target agents often fail to address the distinct pathological features of different CVDs), and uncertain long-term safety (e.g., potential increase in infection risk with anti-inflammatory agents). Furthermore, advances in multi-omics technologies, including single-cell sequencing and spatial transcriptomics, have enabled high-resolution mapping of cell-specific NLRP3 roles in CVDs (e.g., in macrophages, smooth muscle cells, and endothelial cells). The central remaining task is to translate these high-dimensional datasets into validated mechanistic understanding and actionable therapeutic targets.

Building on the clinical and mechanistic evidence outlined above, this review integrates recent advances in the molecular mechanisms of the NLRP3 inflammasome in CVDs and in the development of targeted therapeutics. It first examines how the canonical two-signal model (priming and activation, including inflammasome assembly) operates across major CVD subtypes—atherosclerosis, heart failure, myocardial infarction, and hypertension-related target organ damage—with emphasis on how DAMPs engage NLRP3 in distinct disease microenvironments and drive differential activation of downstream signaling pathways. Next, it surveys intervention strategies directed at the NLRP3 inflammasome, ranging from traditional non-specific anti-inflammatory drugs to modern approaches such as small-molecule inhibitors, antibody drugs, gene-editing technologies, and nanodelivery systems, while comparing their mechanisms of action, preclinical efficacy, and prospects for clinical translation. Finally, drawing on current research gaps, it identifies priority directions for future work, including clarification of NLRP3 crosstalk with other pathways, development of tissue- and cell-specific targeted agents, and construction of multi-omics-based precision classification frameworks for NLRP3-related CVDs. By synthesizing these areas, the review offers an integrated perspective that connects mechanistic understanding with therapeutic development and highlights opportunities for precision strategies in CVD prevention and treatment.

## 2 Biological functions of the NLRP3 inflammasome

The NOD-like receptor pyrin domain-containing 3 (NLRP3) inflammasome is a cytosolic multi-protein complex composed of the NLRP3 sensor protein, apoptosis-associated speck-like protein containing a CARD (ASC), and pro-caspase-1 (19, 20). Excessive or dysregulated activation of the NLRP3 inflammasome is linked to systemic inflammation and contributes to multiple diseases, including gout (21), type 2 diabetes mellitus (22), neurodegenerative diseases (23), atherosclerosis (24), and inflammatory bowel disease (25).

Recent studies have identified NIMA-related kinase 7 (NEK7), a member of the NIMA-related kinase (NEK) family involved in cell-cycle progression. NEK7 comprises a core kinase domain and a short N-terminal tail (26, 27) and is required for mitotic spindle assembly and cytokinesis. Substantial evidence shows that NEK7 binds the leucine-rich repeat (LRR) domain of NLRP3 downstream of potassium efflux, thereby promoting NLRP3 oligomerization and inflammasome activation in a kinase-independent manner. During mitosis, NEK7 also interacts with NEK9, which limits NLRP3 inflammasome activation in interphase cells (27). Notably, NEK7 deficiency in cellular models carrying cryopyrin-associated periodic syndrome (CAPS)-associated NLRP3 mutations impairs caspase-1 activation, whereas the NEK7–mutant NLRP3 interaction is enhanced. Although the dual roles of NEK7 in mitosis and NLRP3 activation are well established, the precise molecular mechanisms of the NLRP3–NEK7 interaction remain to be fully defined.

Canonical inflammasome activation generally converges on caspase-1 recruitment in response to diverse microbial or danger signals. By contrast, the non-canonical inflammasome directly senses cytosolic lipopolysaccharide (LPS) and bacterial mRNA through the CARD domains of caspase-11 (in mice) or caspase-4 and caspase-5 (in humans). This pathway initiates signaling independently of caspase-1, leading to proteolytic maturation of interleukin-1β (IL-1β) and pyroptosis. Recent work demonstrates that caspase-11 can engage the NLRP3 inflammasome through direct interaction between its scaffold domain and the LRR/pyrin domain (PYD) regions of NLRP3 (28), thereby recruiting caspase-1 and promoting maturation of IL-1β and IL-18 (29). Importantly, caspase-11 binds LPS directly via its CARD domain and thus functions as a cytosolic LPS sensor independent of the cell-surface receptor toll-like receptor 4 (TLR4) (28, 30). Because caspase-11 is a key mediator of septic shock, caspase-11-deficient mice are resistant to LPS-induced lethality, underscoring the need to investigate the non-canonical pathway in human cells.

### 2.1 Overview of the NLRP3 inflammasome

The NLRP3 inflammasome is a cytosolic signaling complex containing a nucleotide-binding oligomerization domain (NOD), LRR, and pyrin domain (PYD). It comprises the sensor protein NLRP3, the adaptor ASC, and the effector caspase-1 (19, 31, 32). Upon activation, NLRP3 oligomerizes and recruits ASC, which self-assembles into helical filamentous structures that provide a platform for pro-caspase-1 recruitment and activation. Active caspase-1 then cleaves pro-IL-1β and pro-IL-18 into their mature, secreted forms (33, 34). The NLRP3 inflammasome plays an essential role in innate immune defense against pathogens and cellular damage. However, its aberrant activation drives the pathogenesis of several human diseases, including exacerbation of gouty arthritis, development of CAPS in genetically susceptible individuals, and contribution to inflammatory bowel disease (IBD), obesity, type 2 diabetes mellitus, and Alzheimer’s disease (AD) (35–37). Consequently, pharmacological targeting of NLRP3 represents a promising therapeutic strategy for these conditions.

### 2.2 Activation and regulatory mechanisms of the NLRP3 inflammasome

Most inflammasome sensor proteins recognize distinct pathogen-associated molecular patterns (PAMPs), such as bacterial proteins, toxins, and nucleic acids, thereby triggering assembly during microbial or viral infection. The NLRP3 sensor, however, oligomerizes in response to a broad spectrum of stimuli, including pathogen-derived molecules (e.g., nigericin and bacterial cell-wall components), endogenous danger-associated molecular patterns (DAMPs) (e.g., extracellular ATP, amyloid-β aggregates, and uric acid crystals), and environmental pollutants (e.g., silica, asbestos, and alum) (20). Because of this diversity, NLRP3 is thought to detect common cellular perturbations induced by these activators rather than the ligands themselves. Proposed mechanisms include generation of reactive oxygen species (ROS), release of oxidized mitochondrial DNA (mtDNA), lysosomal destabilization, mitochondrial dysfunction, formation of membrane pores, alterations in intracellular calcium, and potassium efflux (38). Two principal modes of NLRP3 activation have been characterized: canonical and non-canonical.

### 2.3 Lipotoxicity and metaflammation in NLRP3 inflammasome activation

Beyond classical pathogen- and damage-associated molecular patterns, lipotoxicity and metaflammation have emerged as important contributors to NLRP3 inflammasome activation in metabolic cardiovascular diseases. Lipotoxicity, characterized by the excessive accumulation of lipids and their bioactive metabolites such as free fatty acids, ceramides, and cholesterol crystals, can trigger NLRP3 inflammasome activation through mechanisms including lysosomal destabilization, mitochondrial dysfunction, and endoplasmic reticulum stress (39). These lipid species act as endogenous danger signals that link metabolic overload to inflammatory responses.

Metaflammation, referring to the chronic low-grade inflammation driven by metabolic dysregulation, further amplifies NLRP3 activation (40). In conditions of obesity and diabetes, lipid accumulation promotes NLRP3 inflammasome activity in adipose tissue, liver, and cardiovascular tissues (41). This leads to the release of pro-inflammatory cytokines such as IL-1β and IL-18, which in turn exacerbate insulin resistance, endothelial dysfunction, and myocardial inflammation, thereby accelerating the development of cardiovascular complications.

Recent evidence has underscored the close crosstalk between lipid metabolism and NLRP3 inflammasome signaling. Ceramides and saturated fatty acids have been identified as direct activators of the NLRP3 inflammasome (42), whereas interventions that alleviate lipid overload or improve mitochondrial function can suppress NLRP3 activation. These findings suggest that targeting lipotoxicity and metaflammation represents a promising therapeutic approach to modulate NLRP3-driven inflammation in metabolic cardiovascular diseases.

### 2.4 Canonical NLRP3 inflammasome activation

Canonical NLRP3 inflammasome activation requires two sequential and parallel steps: priming (transcription) and oligomerization/assembly (activation) (29). During the priming phase, innate immune signaling—primarily through toll-like receptors (TLRs) and their adaptor myeloid differentiation primary response 88 (MyD88) or through cytokine receptors—activates the nuclear factor-κB (NF-κB) pathway. This upregulates transcription of NLRP3 and pro-IL-1β. In the activation phase, stimuli such as monosodium urate (MSU) crystals, inorganic particles, and bacterial toxins (44) induce ASC speck formation and caspase-1 activation. ASC recruitment is indispensable for cleavage of pro-caspase-1 into its active form. Active caspase-1 then drives pyroptosis and amplifies systemic inflammatory responses (43).

Multiple cellular events converge to promote NLRP3 inflammasome assembly. These include changes in ion fluxes (K^+^, Cl^−^, and Ca^2+^), ROS generation, and lysosomal damage (44). Potassium efflux, triggered by extracellular ATP or nigericin acting on the P2X purinergic receptor 7 (P2X7), activates NEK7, which interacts with the LRR domain of NLRP3 to facilitate inflammasome formation. Mitochondrial dysfunction generates oxidized mtDNA and mitochondrial ROS (mtROS), both of which promote NLRP3 activation. Excessive calcium release from the endoplasmic reticulum can cause mitochondrial calcium overload, mtROS production, and oxidized mtDNA release. Phagocytosis of particulate matter can rupture lysosomal membranes, releasing cathepsin B into the cytosol and further activating NLRP3.

In addition to the canonical pathway, non-canonical activation has been described. This route depends on caspase-4 and caspase-5 in humans or caspase-11 in mice (44). Gram-negative bacteria (e.g., Escherichia coli, Vibrio cholerae, Salmonella typhimurium) activate TLR4–MyD88 and toll/IL-1 receptor homology domain-containing adapter-inducing interferon-β (TRIF) signaling, leading to NF-κB-mediated transcription of IL-1β, IL-18, NLRP3, interferon regulatory factor 7 (IRF7), and IRF3. The IRF7–IRF3 complex induces type I interferon (IFN-α/β) production, which via the janus kinase/signal transducer and activator of transcription (JAK/STAT) pathway upregulates caspase-11 expression (45). In the second phase, an unidentified receptor or scaffold activates caspase-11, which then engages the canonical NLRP3 pathway to release IL-1β and induce pyroptosis. A caspase-8-dependent non-canonical route has also been reported (46–48). In this pathway, DAMPs or PAMPs stimulate TLR4, leading to caspase-8 activation. The receptor-interacting protein 1 (RIP1)–FAS-associated death domain (FADD) complex can contribute to NLRP3 priming and activation steps. In disease settings, accumulation of misfolded proteins and metabolic disturbances can serve as endogenous DAMPs that directly prime the NLRP3 inflammasome.

## 3 NLRP3 inflammasome and cardiovascular diseases

Activation of the NLRP3 inflammasome is closely linked to multiple cardiovascular diseases, although its kinetics differ between acute and chronic injury (48). In acute settings, NLRP3 is rapidly upregulated, narrowing the therapeutic window for intervention. In chronic conditions such as atherosclerosis, hypertension, and heart failure, inhibition at various stages can slow progression and offers a wider therapeutic window. Consequently, targeting the NLRP3 inflammasome represents a potential strategy to mitigate several cardiovascular diseases. This section summarizes current understanding of its roles in atherosclerosis, hypertension, heart failure, and related events (8, 49).

**Figure 1:**
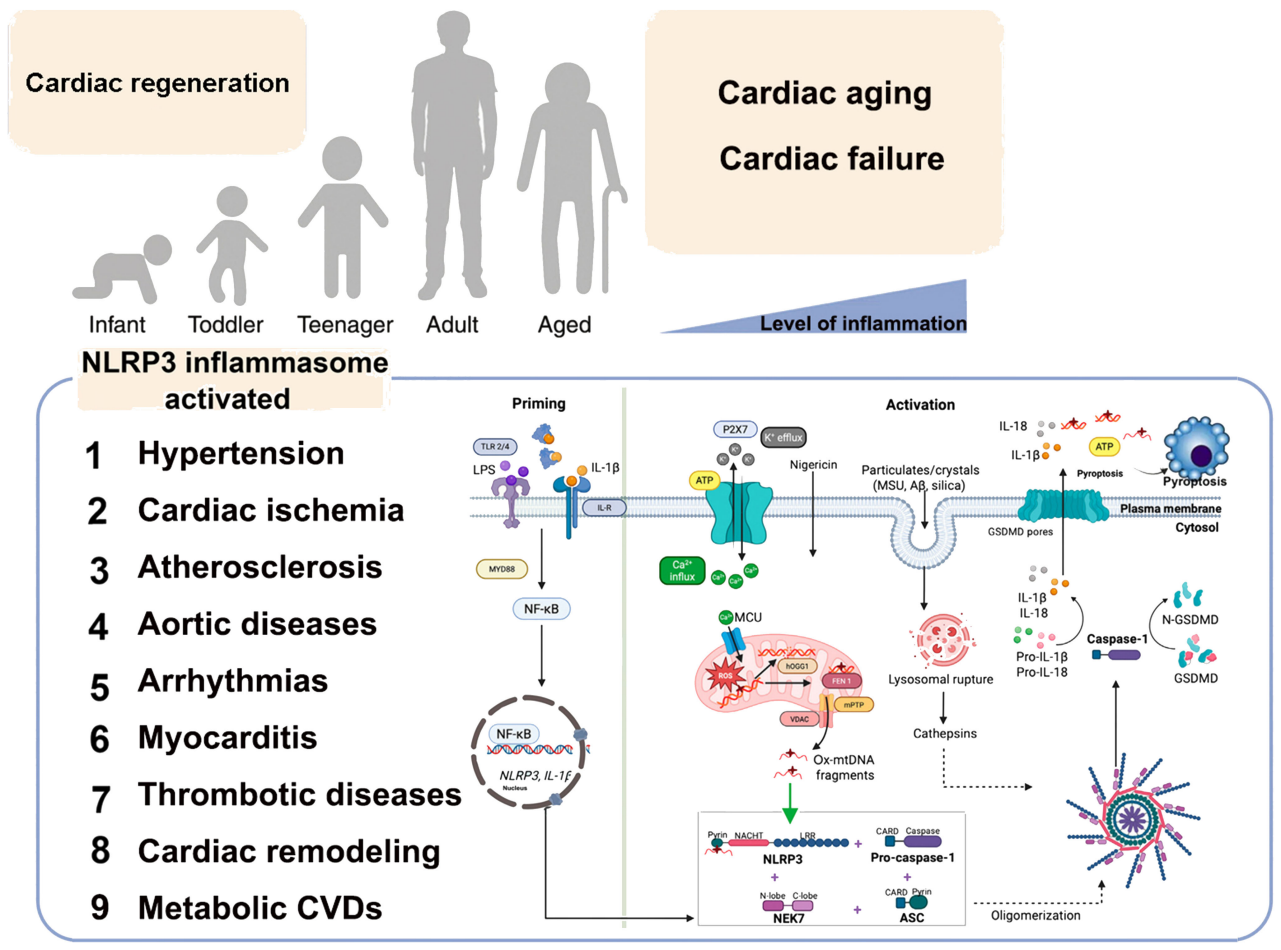
The NLRP3 inflammasome as a master regulator of cardiovascular diseases across the lifespan (This figure was created with BioRender). The schematic depicts the canonical two-step activation of the NLRP3 inflammasome, consisting of priming (via NF-κB signaling triggered by PAMPs/DAMPs) and activation (driven by various cellular stresses such as K^+^ efflux, ROS, lysosomal damage, and mitochondrial dysfunction). Activated NLRP3 inflammasome leads to caspase-1-dependent maturation of IL-1β and IL-18, as well as GSDMD-mediated pyroptosis. The figure also highlights the age-dependent decline in cardiac regenerative capacity and the progressive increase in chronic inflammation (inflammaging), which contribute to the development of multiple cardiovascular conditions, including hypertension, cardiac ischemia, atherosclerosis, aortic diseases, arrhythmias, myocarditis, thrombotic diseases, cardiac remodeling, and metabolic CVDs. This integrated view positions the NLRP3 inflammasome as a promising therapeutic target across the cardiovascular disease spectrum.

A broad overview of the NLRP3 inflammasome as a central regulator across multiple cardiovascular diseases and throughout the lifespan is illustrated in **Figure 1**.

### 3.1 NLRP3 inflammasome in atherosclerosis

#### 3.1.1 Molecular mechanisms

Atherosclerosis is recognized as a chronic inflammatory disease involving recruitment of immune cells to the arterial wall. In early stages, immune cells accumulate in the intima, promoting monocyte recruitment and formation of macrophage-derived foam cells. At the same time, low-density lipoprotein (LDL) is oxidized to oxidized LDL (ox-LDL), which accumulates in the intima and contributes to plaque development (50). Multiple cell types participate, including endothelial cells (ECs), vascular smooth muscle cells (VSMCs), and inflammatory cells.

Endothelial dysfunction and sustained vascular inflammation are key features of atherosclerosis. Stimuli such as microorganisms, crystals, and metabolic disorders can activate the NLRP3 inflammasome in ECs, leading to release of mature IL-1β and IL-18 that further amplify endothelial inflammation and oxidative stress (11). In human umbilical vein endothelial cells (HUVECs) exposed to ox-LDL, interleukin-37 (IL-37) suppresses NLRP3 inflammasome activation, reduces secretion of IL-1β and tumor necrosis factor-α (TNF-α), and attenuates endothelial inflammation and dysfunction (51). Likewise, miR-30c-5p inhibits NLRP3-mediated pyroptosis in human aortic endothelial cells (HAECs) by targeting forkhead box O3 (FOXO3) (52). Endothelial-specific overexpression of forkhead box P1 (Foxp1) in mice reduces monocyte infiltration and lesion formation (53). EC-specific deletion of GATA binding protein 6 (GATA6) similarly decreases monocyte infiltration and plaque formation, partly through regulation of the CMPK2-NLRP3 pathway (54).

VSMCs are essential for maintaining vascular tone and contribute substantially to atherosclerosis. Nicotine activates the NLRP3 inflammasome in VSMCs via lysosomal dysfunction, promoting plaque instability (55). Caspase-8 enhances inflammatory cell infiltration and smooth muscle cell proliferation through NLRP3 and IL-1β signaling (56). Ox-LDL induces VSMC pyroptosis via NLRP3 activation; caspase-1 inhibitors can block this process, indicating that caspase-1 may serve as a therapeutic target. Extracellular NLRP3 inflammasome particles can be internalized by human coronary artery smooth muscle cells, triggering further inflammation and contributing to disease progression, which may represent a novel form of intercellular communication (57). During atherosclerosis, VSMCs can transdifferentiate into macrophage-like and foam cell phenotypes, and NLRP3 expression is elevated in these cells within plaques. Monocytes can drive VSMC phenotypic switching through NLRP3 activation, thereby increasing plaque vulnerability (58). NLRP3 activation in human VSMCs induces secretion of high mobility group box-1 protein (HMGB1), which promotes foam cell formation and accelerates atherosclerosis in ApoE-deficient mice (59). Fibronectin type III domain-containing protein 5 (FNDC5) upregulates NLRP3 and IL-1β via NF-κB signaling, contributing to ox-LDL-induced VSMC foam cell formation and monocyte adhesion (60).

When the fibrous cap thins and becomes infiltrated by inflammatory cells, plaques may undergo calcification and become prone to rupture. Rupture exposes thrombogenic material to the circulation, activating the coagulation cascade through tissue factor, leading to platelet aggregation and superimposed thrombus formation (61).

However, most of the current evidence regarding the role of the NLRP3 inflammasome in atherosclerosis is derived from rodent models, which may not fully recapitulate the complex plaque microenvironment and immune cell dynamics observed in human disease. In addition, the relative contribution of NLRP3 in different cell types—particularly endothelial cells versus macrophages and vascular smooth muscle cells—during different stages of plaque development remains incompletely defined. Furthermore, while many studies have focused on the pro-inflammatory effects of NLRP3, its potential role in plaque stabilization or resolution of inflammation at later stages has received less attention. These uncertainties highlight the need for more refined, cell-specific and stage-specific investigations before NLRP3 can be confidently positioned as a therapeutic target in atherosclerosis.

#### 3.1.2 Natural products targeting the NLRP3 inflammasome in atherosclerosis

A variety of natural products have been shown to modulate NLRP3 inflammasome activity across endothelial cells, vascular smooth muscle cells, and macrophages, thereby exerting protective effects against atherosclerosis (62, 63). These compounds generally act by suppressing NLRP3 expression, inhibiting caspase-1 and ASC activity, and reducing the release of inflammatory mediators and ROS (64, 65).

##### 3.1.2.1 Andrographolide

Andrographolide, the principal active constituent of Andrographis paniculata, inhibits ox-LDL-induced NLRP3 inflammasome assembly in macrophages. This action reduces caspase-1 activation and decreases secretion of IL-1β and IL-18 while limiting excessive ROS production, thereby suppressing foam cell formation and supporting plaque stability (59, 66).

##### 3.1.2.2 Polydatin

Polydatin, a glycosylated derivative of resveratrol, regulates lipid metabolism, suppresses inflammatory mediator release, and enhances antioxidant enzyme activity. In ApoE-deficient mice fed a high-fat diet, it inhibits NLRP3 inflammasome activation and caspase-1 cleavage, exerting protective effects against atherosclerosis through promotion of autophagy and inhibition of pyroptosis (67).

##### 3.1.2.3 Quercetin

Quercetin, a natural flavonoid with antioxidant and anti-inflammatory properties, attenuates atherosclerosis progression in ApoE-deficient mice on a high-fat diet. It inhibits NLRP3 inflammasome activation in ox-LDL-loaded macrophages, reduces cellular steatosis and oxidative stress, and suppresses macrophage pyroptosis (68, 69).

##### 3.1.2.4 Salvianolic acid A and salvianolic acid B

Salvianolic acids A and B, major active components of Salvia miltiorrhiza, protect against atherosclerosis-related inflammation by inhibiting the NF-κB/NLRP3 pathway, reducing ROS release, and modulating glycolysis and pyroptosis. In LDL receptor-deficient mice fed a high-fat diet, salvianolic acid B decreases lesion size via NF-κB/NLRP3 inhibition. *In vitro*, it attenuates TNF-α-induced endothelial inflammation in HUVECs by suppressing ROS production. It also directly binds pyruvate kinase M2 (PKM2), inhibiting the PKM2/PKR pathway in glucose-stimulated HUVECs and thereby reducing expression of caspase-1, gasdermin D (GSDMD), and IL-18 while regulating endothelial pyroptosis in diabetes-associated atherosclerosis (70–72).

##### 3.1.2.5 α-Hydroxyisobutyric acid and related compounds

α-Hydroxyisobutyric acid, a gut microbiota metabolite, increases ROS production in endothelial cells. Paeonol lowers levels of this metabolite, decreases ROS generation, inhibits thioredoxin-interacting protein (TXNIP), and modulates the ROS/TXNIP/NLRP3 pathway, thereby alleviating endothelial inflammation in atherosclerotic mice (73). Paeoniflorin inhibits ox-LDL-induced VSMC proliferation and migration through ERK1/2/MAPK and NF-κB signaling, reduces overexpression of inflammatory cytokines and chemokines, and slows atherosclerosis progression (74).

##### 3.1.2.6 Baicalein

Baicalein, the main flavonoid compound in Scutellaria baicalensis, alleviates inflammation by inhibiting NLRP3 inflammasome assembly and blocking the NLRP3/caspase-1 pathway (75).

##### 3.1.2.7 Oridonin

Oridonin effectively inhibits NLRP3 inflammasome activation in ApoB-deficient mice, thereby reducing inflammatory responses. It also enhances antioxidant capacity in ApoE-deficient mice by increasing the stability and activity of nuclear factor erythroid 2-related factor 2 (Nrf2) and reducing Nrf2 ubiquitination and degradation, suggesting therapeutic potential in atherosclerosis and related diseases (76).

#### 3.1.3 Traditional Chinese medicine formulas targeting the NLRP3 inflammasome in atherosclerosis

Several traditional Chinese medicine formulas and patent medicines have been investigated for their effects on the NLRP3 inflammasome in atherosclerosis. These multi-component preparations often act through multiple targets and pathways, offering advantages in slowing disease progression.

##### 3.1.3.1 Tongxinluo capsule

Tongxinluo Capsule, a traditional Chinese patent medicine used for replenishing qi, activating blood circulation, and relieving pain, has shown anti-atherosclerotic effects associated with inhibition of ROS/NLRP3/caspase-1-mediated endothelial cell pyroptosis (77).

##### 3.1.3.2 Xinmaikang tablet

Xinmaikang Tablet, commonly used for prevention and treatment of atherosclerosis, improves lipid metabolism disorders and inhibits inflammatory responses. Its mechanism may involve suppression of the sterol regulatory element-binding protein 2 (SREBP2)-mediated NLRP3/ASC/caspase-1 signaling axis, thereby reducing secretion of pro-inflammatory cytokines such as IL-6, TNF-α, and vascular cell adhesion molecule 1 (VCAM-1) and attenuating arterial inflammation (78).

##### 3.1.3.3 Zhilong Huoxue Tongyu Capsule

In a New Zealand white rabbit model, Zhilong Huoxue Tongyu Capsule exerts significant lipid-lowering effects and inhibits excessive expression of inflammatory factors including NF-κB, NLRP3, IL-1β, and IL-18 in the carotid artery, effectively improving hyperlipidemia and atherosclerosis (79).

##### 3.1.3.4 Biejia Jianwan

Biejia Jianwan, a traditional Chinese formula that promotes blood circulation to remove stasis and softens hard masses, ameliorates lipid metabolism disorders and vascular endothelial dysfunction in diabetes-related atherosclerosis mice by inhibiting NLRP3 inflammasome activation (80).

##### 3.1.3.5 Huanglian Jiedu Decoction

Huanglian Jiedu Decoction, a classic formula with heat-clearing and detoxifying effects, significantly reduces serum TNF-α and IL-1β levels and decreases NLRP3 inflammasome expression in arterial tissues. It also increases serum IL-10 levels and enhances expression of collagen fibers and α-smooth muscle actin (α-SMA), contributing to improved atherosclerotic plaque stability (81).

##### 3.1.3.6 Qingre Huoxue Decoction

Qingre Huoxue Decoction attenuates atherosclerosis by inhibiting the NF-κB signaling pathway and inducing M2 macrophage polarization. It reduces expression of monocyte chemoattractant protein-1 (MCP-1), NLRP3, and TNF-α in atherosclerotic mice, downregulates M1 polarization markers (inducible nitric oxide synthase (iNOS), C-C chemokine receptor type 7 (CCR7), IL-1β), and upregulates M2 polarization markers (arginase-1 (Arg-1), cluster of differentiation 163 (CD163), chitinase 3-like 3 (Ym-1), found in inflammatory zone 1 (Fizz-1)) (82).

### 3.2 NLRP3 Inflammasome in myocardial infarction

#### 3.2.1 Mechanisms in acute myocardial infarction

Acute myocardial infarction (AMI) is characterized by acute ischemic necrosis of the myocardium due to severe reduction in coronary blood flow. It is the most severe complication of coronary atherosclerosis and usually results from thrombotic occlusion that suddenly deprives the myocardium of oxygen and nutrients, causing cell swelling, rupture, and release of intracellular contents (83). Reperfusion therapy has transformed AMI management; however, restoring blood flow can paradoxically cause additional injury, known as ischemia/reperfusion (I/R) injury. This injury arises from rapid correction of intracellular acidosis, leading to calcium overload, mitochondrial dysfunction, and oxidative stress. These processes also initiate sterile inflammation that can enlarge the infarct and worsen cardiac function. Cellular debris from necrotic myocytes and damage from reperfusion act as damage-associated molecular patterns (DAMPs), amplifying local and systemic inflammatory responses (84).

In AMI, NLRP3 priming and activation are largely driven by cellular debris binding to toll-like receptors, which induces inflammasome formation in cardiomyocytes, leukocytes, fibroblasts, and endothelial cells (85). Priming depends on NF-κB signaling, triggered when DAMPs engage membrane TLRs or IL-1 receptors. This activates myeloid differentiation primary response 88 (MyD88) and interleukin-1 receptor-associated kinases (IRAKs), leading to NF-κB-mediated transcription of NLRP3, ASC, pro-caspase-1, pro-IL-1β, and pro-IL-18. Nucleotide-binding oligomerization domain-containing protein 2 (NOD2) can similarly prime the inflammasome by directly activating NF-κB (86).

NLRP3 activation occurs through extracellular and intracellular routes. Extracellular ATP activates the P2X purinergic receptor 7 (P2X7), causing potassium efflux that is sensed by NEK7, which then binds NLRP3 to promote assembly. Intracellularly, lysosomal destabilization (e.g., by uric acid or cholesterol crystals) releases cathepsin B and further promotes potassium efflux. Mitochondrial dysfunction increases reactive oxygen species (ROS) production, which activates NLRP3 via thioredoxin-interacting protein (TXNIP) and contributes to pyroptosis and infarct expansion (87). Multiple studies have demonstrated that inhibiting the NLRP3 inflammasome reduces inflammation and improves myocardial function and remodeling in AMI models (88).

#### 3.2.2 Mechanisms in ventricular remodeling after acute myocardial infarction

Even with reperfusion, many patients experience irreversible loss of myocardial function, prompting compensatory hypertrophy of remaining cardiomyocytes—a process termed ventricular remodeling. This remodeling is a major cause of progression to end-stage heart failure (89) and involves both exaggerated inflammation and neurohumoral activation as part of a compensatory repair response (90).

NLRP3 inflammasome activation converts pro-caspase-1 to active caspase-1, which cleaves pro-IL-1β and pro-IL-18 into mature forms. Caspase-1 also cleaves gasdermin D (GSDMD) into its active N-terminal fragment (GSDMD-NT), which oligomerizes to form membrane pores. These pores disrupt membrane integrity, causing lytic cell death (pyroptosis) and release of IL-1β and IL-18. The released cytokines recruit neutrophils, activate macrophages, and expand T-cell populations, thereby amplifying inflammation (91).

In mouse AMI models, silencing P2X7 reduces caspase-1 activation, attenuates ventricular remodeling, and improves ventricular dilatation and dysfunction compared with P2X7-expressing controls (92). These findings suggest that NLRP3-mediated pyroptosis may be a viable therapeutic target in post-MI remodeling.

It should be noted, however, that the majority of studies investigating NLRP3-mediated pyroptosis in post-MI remodeling have been conducted in acute or subacute models, with relatively limited data on its role during the chronic phase of ventricular remodeling. Moreover, the extent to which NLRP3 activation in cardiomyocytes versus non-cardiomyocyte populations (such as fibroblasts and immune cells) contributes to adverse remodeling remains unclear. Given that pyroptosis may also participate in the clearance of damaged cells during the early reparative phase, indiscriminate inhibition of NLRP3 could potentially interfere with beneficial repair processes. These considerations underscore the importance of defining optimal intervention windows and cell-specific targeting strategies in future studies.

#### 3.2.3 Therapeutic targeting of the NLRP3 inflammasome in myocardial infarction

The NLRP3 inflammasome has become an attractive target for the treatment of coronary heart disease. Several inhibitors have been investigated in preclinical and clinical studies.

##### 3.2.3.1 MCC950

MCC950 (also known as CRID3 or CP-456773) is a selective small-molecule NLRP3 inhibitor that directly binds the NACHT domain, blocking ATP hydrolysis and preventing inflammasome assembly (93). It has demonstrated protective effects against atherosclerosis in ApoE-deficient mice, but clinical development was discontinued due to hepatotoxicity.

##### 3.2.3.2 BAY11-7082

BAY11–7082 inhibits NLRP3 ATPase activity. In rat models of acute myocardial infarction, pretreatment with BAY11–7082 significantly reduced infarct size and preserved left ventricular function (94).

##### 3.2.3.3 VX-740 and VX-765

VX-740 (pralnacasan) and its analog VX-765 are caspase-1 inhibitors. VX-765 has been shown to reduce ox-LDL-induced VSMC pyroptosis *in vitro* and to inhibit atherosclerosis development in ApoE-deficient mice independently of lipid-lowering effects (95).

##### 3.2.3.4 Colchicine

Colchicine is a non-specific NLRP3 inhibitor. In mouse models of non-reperfused AMI, high-dose colchicine reduced NLRP3 activation, infarct size, and improved ventricular remodeling (96). In a phase II clinical trial, colchicine (0.5 mg once or twice daily for 5 days) was well tolerated and significantly reduced infarct size and inflammatory biomarkers in AMI patients (97). Low-dose colchicine in another trial decreased rehospitalizations for cardiovascular events, although it did not significantly lower CRP levels (98).

##### 3.2.3.5 Canakinumab

Canakinumab is a human monoclonal antibody targeting IL-1β. In the large-scale CANTOS trial involving 10,061 patients with prior myocardial infarction and elevated hsCRP, canakinumab (50, 100, or 150 mg every 3 months) significantly reduced major adverse cardiovascular events compared with placebo (17).

### 3.3 NLRP3 inflammasome in heart failure

Activation of the NLRP3 inflammasome leads to caspase-1 activation and subsequent release of IL-1β and IL-18, which promote myocardial hypertrophy, fibrosis, and ventricular remodeling, thereby accelerating heart failure (HF) progression (99). Active caspase-1 also cleaves GSDMD to induce pyroptosis, contributing to chronic ventricular remodeling (100).

#### 3.3.1 Myocardial hypertrophy

Myocardial hypertrophy is an early compensatory response in HF. Intervening in NLRP3 signaling may therefore offer therapeutic benefit. S-nitrosylation of muscle LIM domain protein promotes formation of a TLR3–receptor-interacting protein kinase 3 complex that activates NLRP3 and accelerates hypertrophy (101). Pharmacological depletion of extracellular ATP or genetic disruption of P2X7 inhibits myocardial NLRP3 activity during pressure overload, indicating that the ATP/P2X7 axis contributes to inflammation and hypertrophy (102). In diabetic cardiomyopathy, hyperlipidemia, hyperglycemia, and insulin resistance induce ROS, NF-κB, and TXNIP, leading to NLRP3 activation (103).

#### 3.3.2 Myocardial fibrosis

Repeated ischemia and hypoxia drive fibroblast proliferation and collagen deposition, resulting in myocardial fibrosis—an important driver of HF progression. Although acute NLRP3 activation may aid repair, chronic activation sustains IL-1β and IL-18 production, promoting fibrosis and adverse remodeling (104). Myocardial cell-specific deletion of CaMKIIδ in transverse aortic constriction (TAC) mice reduces NLRP3 levels, improves ventricular function, and attenuates fibrosis, suggesting CaMKIIδ links pressure overload to NLRP3 activation (105). Norepinephrine receptors can also activate NLRP3 in fibroblasts via ROS and calcium signaling, promoting fibrosis (106). In hypertensive heart disease, the selective serum glucocorticoid-regulated kinase 1 inhibitor EMD638683 blocks NLRP3 activation, reduces IL-1β, inhibits fibroblast-to-myofibroblast transition, and decreases fibrosis (107). After MI, NLRP3-deficient mice show improved survival and infarct healing, indicating that NLRP3 inhibition in the infarct zone may limit progression to HF (108).

#### 3.3.3 Myocardial pyroptosis

Pyroptosis is a pro-inflammatory form of programmed cell death mediated by the NLRP3 inflammasome and contributes to ventricular remodeling (109). Silica nanoparticles increase ROS and activate the NLRP3/caspase-1/GSDMD pathway in cardiomyocytes, inducing pyroptosis (110). Acute β-adrenergic overstimulation activates NLRP3 and triggers pyroptosis; membrane nanotubes can propagate activated inflammasomes to adjacent fibroblasts, amplifying injury (111). In dilated cardiomyopathy, NADPH oxidase 1/4-mediated mitochondrial fission increases ROS and NLRP3 activation, leading to pyroptosis and progressive dysfunction (112). The circular RNA hsa_circ_0076631 (CACR) regulates pyroptosis in diabetic cardiomyopathy cells via the miR-214-3p/caspase-1 pathway (113).

Nevertheless, the precise role of the NLRP3 inflammasome in myocardial fibrosis remains a subject of debate. While chronic NLRP3 activation is generally associated with progressive fibrosis, emerging evidence suggests that it may also contribute to the early reparative response following injury. In addition, most current findings are based on global or cardiomyocyte-specific knockout models, and the contribution of NLRP3 in cardiac fibroblasts versus immune cells during fibrotic remodeling has not been fully delineated. Furthermore, the interplay between NLRP3-driven pyroptosis and other forms of regulated cell death, such as apoptosis and ferroptosis, in the context of heart failure is still poorly understood. These gaps limit our ability to design targeted interventions that selectively suppress maladaptive remodeling without compromising repair mechanisms.

#### 3.3.4 Clinical research progress

Clinical evidence linking the NLRP3 inflammasome to HF remains limited but suggestive. Short-term IL-1 receptor antagonist administration in HF patients improved oxygen consumption, exercise tolerance, and reduced IL-1β levels (114). Exercise training in HF patients increased ASC methylation and decreased IL-1β and ASC mRNA expression, suggesting epigenetic benefits (115). Serum IL-1β and IL-18 levels are elevated in HF and correlate with disease severity, NT-proBNP, and reduced LVEF (116). IL-1 inhibitors have shown favorable effects on remodeling in early trials of both HFpEF and HFrEF (117). However, most data derive from animal models, and factors such as age, sex, comorbidities, and concomitant medications must be considered in future human studies.

#### 3.3.5 Application of NLRP3 inflammasome inhibitors in heart failure

Several agents targeting the NLRP3 inflammasome or its downstream components have been explored for heart failure (HF).

##### 3.3.5.1 MCC950

MCC950 is a selective NLRP3 inhibitor. In transverse aortic constriction (TAC) models, it improved left ventricular ejection fraction (LVEF) and fractional shortening, reduced ventricular dilatation and hypertrophy-related gene expression, and attenuated myocardial fibrosis (118, 119). It has also been shown to reverse left ventricular hypertrophy and improve cardiac structural and electrical remodeling in HF models.

##### 3.3.5.2 OLT1177

OLT1177 specifically inhibits the NLRP3 inflammasome by blocking ATPase activity and reducing IL-18 and IL-1β secretion. In a mouse model of non-reperfused anterior wall myocardial infarction, it preserved β-adrenergic receptor reactivity and prevented left ventricular systolic and diastolic dysfunction (120). In a phase 1B double-blind trial in patients with HFrEF, 14 days of OLT1177 treatment significantly improved LVEF and exercise time with good tolerability (121).

##### 3.3.5.3 Colchicine

Colchicine inhibits NLRP3 inflammasome activation through interference with P2X7R–ASC interactions and mitochondrial transport. It has been shown to improve cardiac function, exercise tolerance, and ventricular remodeling in relevant models (122, 123). Results in STEMI patients have been mixed, possibly influenced by timing and dosing (124, 125).

##### 3.3.5.4 Empagliflozin

As an antidiabetic agent, empagliflozin inhibits NLRP3 inflammasome activation in HFpEF models in a calcium mobilization-dependent manner, thereby alleviating cardiac insufficiency (126).

##### 3.3.5.5 Triptolide and pirfenidone

Triptolide and pirfenidone, used for fibrosis treatment, inhibit NLRP3 inflammasome activity, reduce macrophage infiltration and fibrosis, and improve both diastolic and systolic dysfunction in TAC-induced HF models (127, 128).

##### 3.3.5.6 VX-765

VX-765 is a highly selective caspase-1 inhibitor. When combined with a P2Y12 receptor antagonist in rat ischemia-reperfusion models, it further reduced infarct size and improved cardiac insufficiency (129).

##### 3.3.5.7 Canakinumab

In sub-analyses of the CANTOS trial, the IL-1β inhibitor canakinumab reduced HF hospitalizations and HF-related mortality (130).

##### 3.3.5.8 Anakinra

Anakinra, an IL-1 receptor antagonist, when administered at discharge in patients with acute decompensated systolic HF, was associated with reduced NT-proBNP levels, improved cardiopulmonary function, and enhanced quality of life at 12 weeks (131) It has also been linked to reduced mortality and HF incidence in STEMI patients (132).

##### 3.3.5.9 IL-18 inhibition

In mouse models, IL-18 gene deletion or anti-IL-18 antibodies reduced cardiac injury and improved function and remodeling under β-adrenergic overstimulation (133). Recombinant IL-18 binding protein improved cardiac contractility and reduced myocardial injury markers in ex vivo resuscitated hearts (134).

Inhibitors of caspase-1, IL-1β, and IL-18 can block NLRP3 inflammasome-mediated effects but may also influence other inflammatory pathways. Their impact on host defense and potential side effects require careful evaluation in future clinical studies.

### 3.4 NLRP3 Inflammasome in atrial fibrillation

Atrial fibrillation (AF) is one of the most common sustained arrhythmias and is associated with substantial morbidity, mortality, and complications such as heart failure, cardiomyopathy, and stroke (135). Current treatments—including antiarrhythmic drugs, rate-control agents, anticoagulation, and catheter ablation—have limited efficacy and do not fully address clinical needs. Consequently, clarifying the molecular mechanisms of AF initiation and maintenance and developing new therapeutic approaches remain important research priorities (136). Although the link between inflammation and AF was proposed more than two decades ago, recent evidence indicates that atrial myocytes themselves express a complete inflammatory signaling machinery. In particular, abnormal activation of the NLRP3 inflammasome appears to contribute to the development and progression of AF (137). This finding offers a new perspective on AF pathogenesis and highlights inflammatory signaling as a potential therapeutic target.

#### 3.4.1 Association with AF pathogenesis

Multiple lines of evidence link the NLRP3 inflammasome to AF. Yao et al. reported elevated NLRP3 protein expression in atrial myocytes from patients with AF and in animal models, including a canine rapid atrial pacing model and a cAMP response element modulator (CREM) transgenic mouse model of spontaneous AF (138). In right atrial tissue from patients with persistent AF, levels of NLRP3, ASC, and active caspase-1-p20 were increased. In CREM transgenic mice, upregulation of NLRP3, ASC, and caspase-1 preceded the onset of spontaneous AF and was accompanied by increased atrial ectopic activity. Genetic inhibition of NLRP3 prevented AF in these mice, supporting a mechanistic connection.

In patients with chronic kidney disease (CKD), Song et al. observed enhanced NLRP3 activity in atrial tissue and elevated circulating IL-1β, particularly in those who also had AF, suggesting that increased NLRP3 activity may contribute to the higher AF risk in CKD (139). Scott et al. found that obesity was associated with elevated atrial NLRP3 levels in humans, sheep, and mice. High-fat diet feeding increased AF susceptibility in wild-type mice, whereas NLRP3 knockout mice showed partial resistance (140). In addition, gut microbiota dysbiosis has been linked to AF, with microbial components or metabolites potentially modulating NLRP3 activity and thereby influencing AF occurrence (141).

#### 3.4.2 Electrical remodeling

Atrial electrical remodeling, characterized by ion channel alterations and changes in ion currents, is a key trigger for AF. The NLRP3 inflammasome can promote electrical remodeling by affecting Ca^2+^ and K^+^ handling (142, 143).

In cardiomyocyte-specific NLRP3 knock-in (CM-KI) mice, Yao et al. observed a high incidence of atrial ectopic rhythms and pacing-induced AF, effects that were attenuated by the NLRP3 inhibitor MCC950 (138). Hyperactive NLRP3 signaling increased ryanodine receptor 2 (RyR2) expression, leading to abnormal sarcoplasmic reticulum Ca^2+^ release and delayed afterdepolarizations. It also upregulated Kcna5 transcription, increasing the ultrarapid delayed rectifier potassium current (IKur) and shortening the atrial effective refractory period (AERP), thereby facilitating reentry (138). AAV9-mediated atrial myocyte-specific NLRP3 knockdown reversed these ion channel changes and suppressed AF (138).

In a CKD model, Song et al. showed that wild-type CKD mice had higher pacing-induced AF susceptibility, longer AF episodes, and shorter action potential duration (APD) and AERP compared with sham controls (139). These changes were attenuated in NLRP3 knockout CKD mice. Kv1.5 protein was upregulated in wild-type CKD atria but normalized in NLRP3-deficient CKD mice, indicating that NLRP3-mediated Kv1.5 upregulation is a major contributor to electrical remodeling in this setting (139). Scott et al. similarly found that obesity increased Kv1.5 expression and shortened AERP in association with elevated NLRP3; NLRP3 inhibition reversed these effects (140). Heijman et al. reported activation of the NLRP3/CaMKII pathway in atrial myocytes from patients with postoperative AF, with increased CaMKII expression, RyR2 hyperphosphorylation, enhanced spontaneous Ca^2+^ leak, and delayed afterdepolarizations (144).

#### 3.4.3 Structural remodeling

Atrial structural remodeling, including dilation, interstitial fibrosis, and extracellular matrix deposition, promotes heterogeneous conduction and reentry that sustain AF. The NLRP3 inflammasome may contribute to fibrosis by activating cardiac fibroblasts.

In CM-KI mice, Yao et al. observed atrial enlargement and increased atrial weight-to-tibial length ratio, together with elevated fibrosis markers (collagen-1a and galectin-3), suggesting that NLRP3-driven fibroblast activation promotes atrial fibrosis and remodeling (138). Zhang et al. linked age-related gut microbiota dysbiosis to AF via intestinal barrier dysfunction, elevated circulating LPS, and enhanced atrial NLRP3 activity (141). This activated the TGF-β1/α-smooth muscle actin (α-SMA) pathway through IL-1β, promoting fibrosis and increasing AF susceptibility; the effect was reversible with young microbiota transplantation (141). Zuo et al. found reduced short-chain fatty acid (SCFA) levels in AF patients and showed that dietary fiber deficiency upregulated NLRP3, caspase-1, and IL-1β, aggravated fibrosis, enlarged the left atrium, and increased AF susceptibility—effects that were improved by SCFA supplementation via the GPR43/NLRP3 pathway (145).

Song et al. further showed that elevated IL-1β in CKD mice correlated with cardiac fibrosis, atrial enlargement, and AF; these changes were prevented in NLRP3 knockout CKD mice (139). Wan et al. demonstrated that IL-1β directly stimulates collagen synthesis and promotes release of pro-fibrotic factors (TNF-α, IL-6, TGF-β1), acting in concert on fibroblasts to increase collagen expression and induce cardiomyocyte hypertrophy (146).

However, the causal relationship between NLRP3 inflammasome activation and atrial structural remodeling in AF remains incompletely established. While multiple studies have demonstrated increased NLRP3 activity in fibrotic atria, it is still unclear whether NLRP3 is a primary driver of fibrosis or a secondary consequence of existing structural abnormalities and hemodynamic stress. Moreover, the majority of mechanistic insights have been obtained from small animal models, which may not accurately reflect the chronic, multifactorial nature of human atrial fibrillation. In addition, the potential interplay between NLRP3-mediated inflammation and other pro-fibrotic pathways, such as TGF-β/Smad and renin-angiotensin-aldosterone system signaling, requires further systematic investigation. These limitations should be taken into account when considering NLRP3 as a therapeutic target for atrial structural remodeling.

#### 3.4.4 Cardiac autonomic nervous regulation

Autonomic nervous system (ANS) imbalance is an important pathophysiological factor in AF. The NLRP3 inflammasome is regulated by sympathetic and vagal activity, and excessive sympathetic activation can drive NLRP3-dependent inflammation.

Isoproterenol (ISO), a non-selective β-adrenoceptor agonist, increases cardiac expression of pro-inflammatory cytokines including IL-1β and IL-18. Knockout of IL-18 or upstream NLRP3 significantly reduced ISO-induced inflammation and macrophage infiltration (147). Phenylephrine activation of α1-adrenoceptors similarly increased cardiac caspase-1, NLRP3, and IL-18 expression, leading to dysfunction and inflammation (148). Blocking β-adrenoceptor signaling inhibited IL-1β, NLRP3, and IL-18 activation and prevented myocardial fibrosis (149). These findings indicate that excessive sympathetic drive promotes AF-related remodeling through NLRP3 inflammasome activation. Strategies that restrain excessive sympathetic activity while enhancing vagal tone may therefore offer new avenues for intervention.

#### 3.4.5 Therapeutic targeting of the NLRP3 inflammasome in atrial fibrillation

Given the role of the NLRP3 inflammasome in AF pathogenesis, it represents a potential therapeutic target. Although clinical data remain limited, several agents have shown promise in preclinical and early clinical studies.

##### 3.4.5.1 MCC950

MCC950 is a potent and selective NLRP3 inhibitor that blocks ATPase activity in the NACHT domain, preventing inflammasome assembly. In CM-KI mice, MCC950 reduced both spontaneous and pacing-induced AF, supporting its potential as a novel therapeutic strategy (138). Clinical translation will require further safety and efficacy evaluation.

##### 3.4.5.2 Colchicine

Colchicine inhibits microtubule polymerization and NLRP3 inflammasome assembly. It has been investigated for prevention of AF recurrence after pulmonary vein isolation and for reduction of postoperative AF (150–152). Clinical trials have yielded encouraging but mixed results, warranting further study.

##### 3.4.5.3 Resolvin D1

RvD1, an endogenous pro-resolving mediator derived from ω-3 polyunsaturated fatty acids, inhibits endoplasmic reticulum stress-induced NLRP3 activation and NF-κB signaling in cardiac tissue. It has been shown to attenuate electrical and structural remodeling and reduce AF occurrence in rat models of post-MI left ventricular dysfunction (153, 154).

##### 3.4.5.4 Salvianolate

Salvianolate, derived from Salvia miltiorrhiza, improved cardiac function, reduced AF duration and P-wave duration, and reversed left atrial enlargement and interstitial fibrosis in a post-MI mouse model. Proposed mechanisms include inhibition of the TGF-β1/Smad2/3 pathway and the TXNIP/NLRP3 pathway (155).

##### 3.4.5.5 P2X7 receptor inhibitors

The P2X7 receptor is an important upstream activator of the NLRP3 inflammasome. Inhibitors of P2X7 have shown preventive effects against AF in rodent models of depression and sterile pericarditis (156, 157).

##### 3.4.5.6 TRPV4 inhibitors

Transient receptor potential vanilloid 4 (TRPV4) is a non-selective cation channel. Blockade of TRPV4 inhibited NLRP3 inflammasome activation via the ERK/NF-κB pathway and reduced AF occurrence in a mouse sterile pericarditis model (158).

##### 3.4.5.7 Extracellular vesicles

Cardiac cell-derived EVs carry bioactive molecules and can modulate intercellular communication. Direct injection of human cardiac-derived EVs into the atrial myocardium of rats with sterile pericarditis reduced atrial fibrosis, lowered inflammatory markers, and decreased AF incidence in a dose-dependent manner, an effect linked to NLRP3 inhibition. EVs from bone marrow or umbilical cord blood did not produce similar benefits (159).

Although progress has been made, several challenges remain. The inflammatory response functions as a double-edged sword; most current NLRP3 inhibitors act systemically and may impair host defense. Therefore, developing agents that preferentially target atrial or local NLRP3 activity, together with well-designed clinical trials, will be essential for translating these findings into effective AF therapies.

### 3.5 NLRP3 inflammasome in diabetic cardiomyopathy

The pathophysiological mechanisms linking type 2 diabetes mellitus (T2DM) and heart failure include myocardial metabolic alterations, impaired energy metabolism, systolic dysfunction, glucotoxicity, lipotoxicity, oxidative stress, myocardial fibrosis and hypertrophy, and diastolic dysfunction. These processes drive myocardial remodeling, systolic and diastolic dysfunction, and microangiopathy, ultimately progressing to heart failure (160). A substantial proportion of patients with T2DM also develop asymptomatic cardiac structural and functional abnormalities (160).

#### 3.5.1 Mitochondria-ROS-NLRP3 inflammasome axis

Mitochondria are central to cardiomyocyte energy metabolism. Mitochondrial dysfunction—characterized by disrupted fusion-fission dynamics, abnormal energy metabolism, excessive reactive oxygen species (ROS) production, altered cardiolipin metabolism, and calcium imbalance—contributes to diabetic cardiomyopathy (DCM). Damaged mitochondria release mitochondrial DNA (mtDNA) and mitochondrial ROS (mtROS), which act as damage-associated molecular patterns (DAMPs) to activate the NLRP3 inflammasome (161). Hyperglycemia markedly increases mtROS in cardiomyocytes, triggering NLRP3 activation and IL-1β secretion. Inhibitors of mitochondrial ROS production, such as Mito-TEMPO or N-acetylcysteine (NAC), significantly suppress NLRP3 activation and IL-1β release (162). These findings indicate that hyperglycemia-induced mitochondrial damage and mtROS overproduction serve as a key upstream signal for NLRP3 inflammasome activation in DCM.

#### 3.5.2 TXNIP-NLRP3 inflammasome axis

Under basal conditions, thioredoxin-interacting protein (TXNIP) binds to thioredoxin (TRX) and remains inactive. When mtROS levels rise, the TXNIP-TRX complex dissociates, allowing free TXNIP to bind the leucine-rich repeat (LRR) domain of NLRP3 and activate the inflammasome (163). In cardiac tissue of diabetic rats, TXNIP, NLRP3 components, and IL-1β levels are markedly elevated compared with non-diabetic controls. Silencing TXNIP in diabetic cardiomyocytes inhibits NLRP3 activation and reduces hyperglycemia-induced cellular injury (164). Thus, TXNIP functions as an important link between hyperglycemic stress and NLRP3 inflammasome activation in DCM.

#### 3.5.3 ROS-NF-κB-NLRP3 inflammasome signaling pathway

Nuclear factor-κB (NF-κB) is a central regulator of inflammatory responses and induces expression of pro-inflammatory cytokines such as TNF-α, IL-6, pro-IL-1β, and pro-IL-18. Hyperglycemia activates NF-κB in cardiomyocytes; elevated ROS promotes NF-κB phosphorylation and nuclear translocation, which upregulates NLRP3 transcription and protein expression (165). In DCM models, increased NF-κB phosphorylation coincides with robust NLRP3 inflammasome activation. The ROS scavenger NAC reduces NF-κB phosphorylation, downregulates NLRP3 expression, and inhibits inflammasome activity. Direct NF-κB inhibition in diabetic rats similarly suppresses NLRP3 inflammasome activation, alleviates cardiac inflammation and fibrosis, and improves cardiac function (165).

Impaired autophagy in diabetic hearts fails to clear damaged mitochondria efficiently, leading to sustained ROS release and NLRP3 inflammasome assembly. In addition, failure to clear activated inflammasomes permits excessive downstream inflammatory signaling and exacerbates myocardial injury (166–168).

#### 3.5.4 NLRP3 inflammasome-mediated myocardial injury and fibrosis

Myocardial fibrosis involves excessive extracellular matrix deposition, increased left ventricular stiffness, and eventual cardiac dysfunction. NLRP3 inflammasome activation is enhanced in cardiac fibroblasts of diabetic rats. High-glucose stimulation upregulates caspase-1 and induces pyroptosis in H9c2 cardiomyocytes; NLRP3 gene silencing significantly reduces pyroptosis and myocardial fibrosis while improving cardiac function in diabetic rats (169). Advanced glycation end products (AGEs) promote cardiomyocyte hypertrophy via the NF-κB-NLRP3-IL-1β pathway, and NLRP3 knockout attenuates angiotensin II-induced hypertrophy (170).

In the hyperglycemic microenvironment, ROS and the P2X7 receptor (P2X7R) synergistically activate NLRP3, promoting collagen synthesis in cardiac fibroblasts (171). H3 relaxin attenuates P2X7R-mediated NLRP3 activation under high-glucose conditions and inhibits collagen synthesis (172). NLRP3 inflammasome activation also suppresses cAMP release in fibroblasts, impairing contractility (173). Both IL-1β and IL-18 stimulate collagen expression in a dose-dependent manner (174).

Transforming growth factor-β1 (TGF-β1) upregulates NLRP3 in cardiac fibroblasts. NLRP3 further promotes fibrotic signaling through a cytokine-independent mechanism by enhancing R-Smad activation and fibroblast differentiation via its NACHT domain. Cardiac fibroblasts lacking NLRP3 show impaired myofibroblast differentiation and reduced R-Smad activation in response to TGF-β (175).

It is important to recognize that current understanding of NLRP3 involvement in diabetic cardiomyopathy is largely based on rodent models of type 1 or type 2 diabetes, which may not fully capture the metabolic complexity and long disease duration typical of human diabetic cardiomyopathy. In addition, while NLRP3 has been implicated in both cardiomyocyte pyroptosis and fibroblast activation, the relative contribution of these two processes to overall cardiac dysfunction at different stages of the disease remains unclear. Furthermore, the crosstalk between NLRP3 and other diabetes-related pathways, such as advanced glycation end products, lipotoxicity, and impaired autophagy, has not been comprehensively characterized. These unresolved issues highlight the need for more integrative and clinically relevant studies before NLRP3 can be considered a viable therapeutic target in diabetic cardiomyopathy.

#### 3.5.5 Therapeutic targeting of the NLRP3 inflammasome in diabetic cardiomyopathy

Excessive NLRP3 inflammasome activation contributes to myocardial inflammation, cardiomyocyte death, fibrosis, and dysfunction in DCM, making it a potential therapeutic target.

##### 3.5.5.1 Metformin

Metformin activates AMP-activated protein kinase (AMPK) and its downstream target peroxisome proliferator-activated receptor α (PPARα). This reduces oxidative stress and ROS release, inhibits NLRP3-mediated inflammation, decreases cardiomyocyte apoptosis, and improves ventricular remodeling and cardiac function in DCM models (176).

##### 3.5.5.2 H3 relaxin

H3 relaxin reduces cardiomyocyte apoptosis and myocardial fibrosis in diabetic rats while directly inhibiting NLRP3 inflammasome activation (177).

##### 3.5.5.3 Coriolus versicolor extract

Extracts from *Coriolus versicolor* attenuate NLRP3 inflammasome activation by inhibiting the NF-κB pathway, reduce caspase-1, IL-1β, IL-18, and NLRP3 components in diabetic rat hearts, alleviate cardiac inflammation, and improve function. They also mitigate fibrosis via inhibition of the TGF-β1/Smad pathway (178).

##### 3.5.5.4 Gypenosides

Gypenosides from *Gynostemma pentaphyllum* inhibit hyperglycemia-induced NLRP3 inflammasome activation and IL-1β/IL-18 secretion by reducing mitochondrial ROS in H9c2 cells and DCM mouse models, thereby ameliorating myocardial injury (179).

##### 3.5.5.5 Quercetin

Quercetin, a flavonoid present in several traditional Chinese medicinal herbs, inhibits inflammasome protein expression, reduces myocardial interstitial fibrosis and cardiomyocyte apoptosis, and alleviates hyperglycemia-induced cardiac dysfunction in diabetic rats (180).

##### 3.5.5.6 Naringenin

Naringenin downregulates NLRP3, caspase-1, ASC, IL-1β, type I collagen, and fibronectin in cardiac tissue, reduces cardiomyocyte apoptosis, ameliorates fibrosis, and inhibits myocardial remodeling in DCM models (181).

##### 3.5.5.7 Ursolic acid

Ursolic acid ameliorates myocardial pathological changes (disorganized fibers, inflammatory infiltration, hypertrophy, and necrosis) in T2DM mouse models through inhibition of NLRP3 inflammasome activation and reduced IL-1β expression (182).

##### 3.5.5.8 Soapnut saponins

Soapnut saponins suppress caspase-1 and IL-1β levels in high-glucose-stimulated H9c2 cells and protect cardiomyocytes by regulating the NLRP3-caspase-1-IL-1β axis (183).

In summary, the NLRP3 inflammasome contributes to the pathogenesis of multiple cardiovascular diseases discussed in this chapter, including atherosclerosis, myocardial infarction, heart failure, atrial fibrillation, and diabetic cardiomyopathy. It promotes inflammation, pyroptosis, and fibrosis through both canonical and non-canonical pathways, ultimately leading to cardiac insufficiency. Although pharmacological targeting of the NLRP3 inflammasome shows promise, further research is needed to develop more selective agents and to conduct well-designed clinical trials.

**Figure 2:**
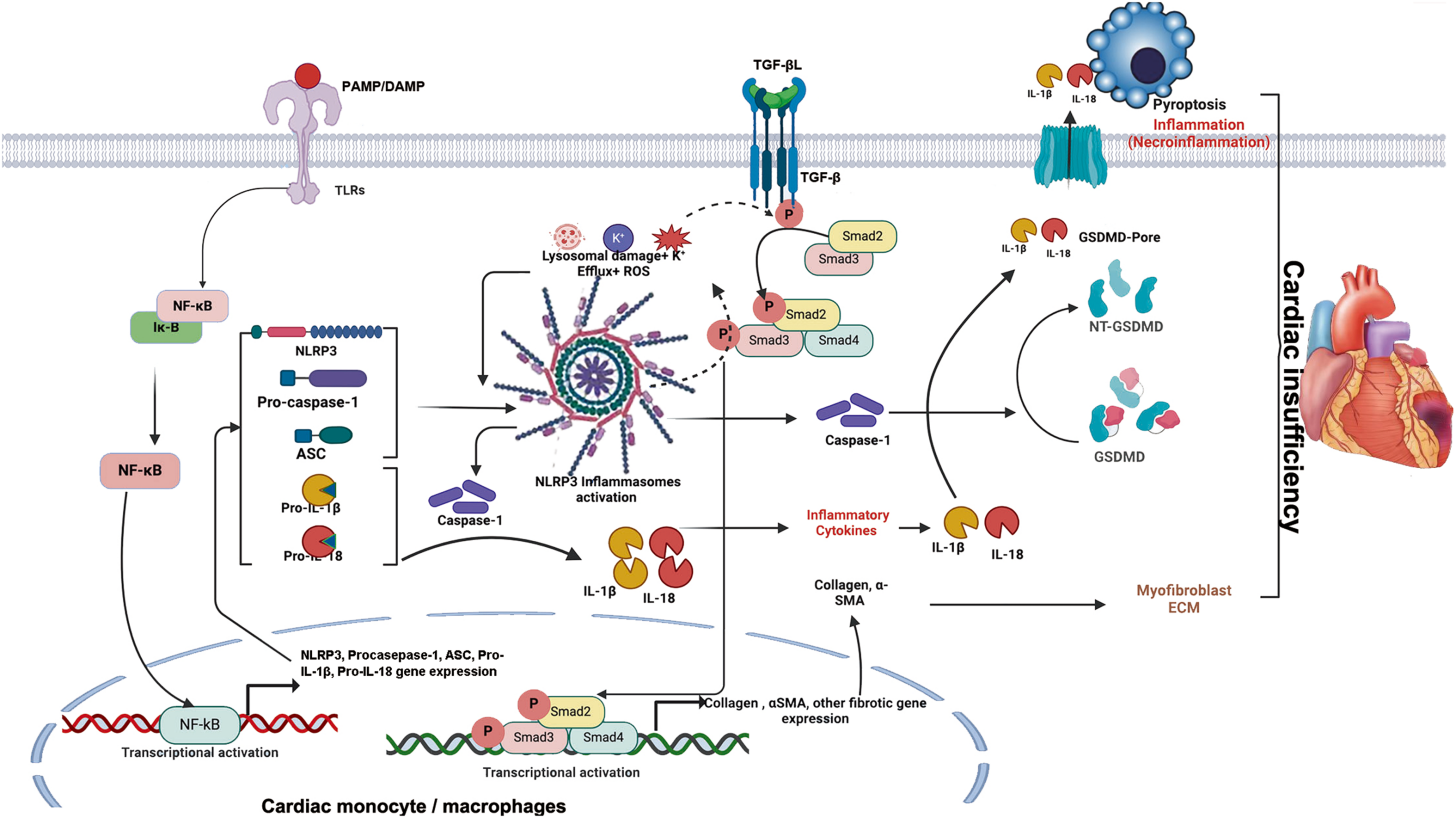
The NLRP3 inflammasome–TGF-β/Smad crosstalk in cardiac monocytes/macrophages mediates cardiac insufficiency [This figure was created with BioRender. Figures adapted from Luo et al., 2025 (184), licensed under CC BY 4.0]. Pathogen-associated molecular patterns (PAMPs) and damage-associated molecular patterns (DAMPs) activate toll-like receptors (TLRs), leading to NF-κB-mediated priming of the NLRP3 inflammasome and upregulation of pro-IL-1β, pro-IL-18, and other components. Cellular stressors, including lysosomal damage, reactive oxygen species (ROS), and potassium (K^+^) efflux, trigger NLRP3 inflammasome assembly and caspase-1 activation. Active caspase-1 cleaves pro-IL-1β and pro-IL-18 into mature pro-inflammatory cytokines and processes gasdermin D (GSDMD) to form membrane pores, resulting in pyroptosis and amplification of inflammation. Concurrently, TGF-β signaling activates Smad2/3/4, promoting the transcription of fibrotic genes (collagen, α-smooth muscle actin [α-SMA]) and driving myofibroblast differentiation with extracellular matrix (ECM) deposition. The integration of NLRP3 inflammasome-driven inflammation and TGF-β/Smad-mediated fibrosis ultimately contributes to cardiac insufficiency.

A common mechanism by which the NLRP3 inflammasome in cardiac monocytes/macrophages drives cardiac insufficiency through integrated inflammatory and fibrotic responses is illustrated in **Figure 2**.

## 4 Therapeutic targets and inhibitors of the NLRP3 inflammasome

The NLRP3 inflammasome signaling pathway can be targeted at multiple levels, including inhibition of NLRP3 itself, ASC oligomerization, caspase-1 activation, and GSDMD-mediated pyroptosis.

### 4.1 Inhibition of the NF-κB pathway

Parthenolide inhibits NF-κB activity and has been shown to improve survival in septic shock models, slow atherosclerotic lesion development in ApoE-deficient mice, and reduce bladder inflammation in cystitis models (185). In addition to its effects on NF-κB, parthenolide directly inhibits multiple inflammasomes by suppressing caspase-1 activity and NLRP3 ATPase function. BAY 11–7082 shows greater selectivity for the NLRP3 pathway. As an IκB kinase inhibitor, it prevents IκBα phosphorylation and alkylates cysteine residues in the NLRP3 ATPase domain, impairing its activity (186). BAY 11–7082 has demonstrated efficacy in models of experimental autoimmune encephalomyelitis and diabetic nephropathy.

β-Hydroxybutyrate (BHB), a ketone body elevated during high-intensity exercise or ketogenic diets, inhibits NLRP3 activation by blocking potassium efflux and ameliorates NLRP3-dependent conditions such as familial cold autoinflammatory syndrome, Muckle-Wells syndrome, and uric acid crystal-induced peritonitis (187). BHB also exerts inhibitory effects on NF-κB signaling.

### 4.2 Inhibition of IL-1β signaling

IL-1β is the principal pro-inflammatory cytokine released upon inflammasome activation. Blocking IL-1β or its receptor represents an effective strategy for treating cryopyrin-associated periodic syndromes (CAPS) and other NLRP3-driven chronic inflammatory diseases (188). Three IL-1-neutralizing biologics have been approved: anakinra, canakinumab, and rilonacept (189). Anakinra was the first agent approved for CAPS and is also used in rheumatoid arthritis. The CANTOS trial showed that canakinumab reduces major adverse cardiovascular events in high-risk patients and alleviates arthritis and gout (190). Rilonacept is approved for recurrent pericarditis in addition to CAPS. These successes validate targeting IL-1 signaling downstream of the NLRP3 inflammasome. However, systemic IL-1 blockade increases infection risk, and anakinra and canakinumab have limited blood-brain barrier penetration (191). Thus, while effective, this approach lacks specificity for NLRP3-driven pathology.

### 4.3 Inhibition of ASC speck formation

ASC oligomerization mediates the interaction between NLRP3 and pro-caspase-1. Inhibiting ASC speck formation can therefore suppress NLRP3 inflammasome activation. JC124 is a NLRP3 inhibitor (IC50 = 3.25 μM in mouse macrophages) that targets ASC oligomerization. It directly binds the NLRP3 complex without affecting ATPase activity and has been shown to downregulate NLRP3, ASC, IL-1β, TNF-α, iNOS, and caspase-1 in a traumatic brain injury model (192).

Isoliquiritigenin, a flavonoid from Glycyrrhiza glabra, reduces serum lipids and insulin in T2DM models and alleviates adipose inflammation by inhibiting ASC oligomerization (193). However, it also inhibits the TLR4/MD-2 complex and IKK, thereby affecting NF-κB signaling (194). Its lack of specificity for the NLRP3 inflammasome limits its utility as a targeted agent.

### 4.4 Inhibition of caspase-1

Caspase-1 is the effector protease responsible for IL-1β/IL-18 maturation and GSDMD cleavage. Pralnacasan (VX-740) and VX-765 are peptidic caspase-1 inhibitors that showed promise in preclinical studies but were discontinued in clinical development due to toxicity concerns (195, 196). Optimization of caspase-1 inhibitors remains an active area of research.

### 4.5 Direct inhibition of the NLRP3 inflammasome

Direct targeting of NLRP3 itself offers a more specific strategy than inhibiting upstream or downstream components. Below we summarize key small-molecule inhibitors and their disease relevance.

#### 4.5.1 MCC950

MCC950 (also known as CP-456,773 or CRID3) is a highly potent and selective NLRP3 inhibitor (IC50 ≈ 7.5–8.1 nM). It blocks both canonical and non-canonical NLRP3 activation without affecting AIM2, NLRC4, or NLRP1 inflammasomes or TLR signaling. MCC950 prevents ASC oligomerization but does not interfere with potassium efflux or calcium influx. It has shown efficacy in models of experimental autoimmune encephalomyelitis, CAPS, non-alcoholic fatty liver disease, and Hutchinson-Gilford progeria syndrome (197, 198). Although a phase II trial in rheumatoid arthritis was discontinued due to hepatotoxicity, MCC950 remains the most widely used reference compound for studying NLRP3 inhibition.

#### 4.5.2 CY-09

CY-09 is an analog of CFTR(inh)-172 that specifically binds the ATP-binding motif in the NACHT domain of NLRP3 and inhibits its ATPase activity (199). It suppresses IL-1β secretion in LPS-primed macrophages stimulated by MSU, nigericin, or ATP, without affecting TNF-α or pro-IL-1β expression. CY-09 has demonstrated activity in synovial cells from gout patients and therapeutic effects in mouse models of CAPS, T2DM, obesity, and Mowat-Wilson syndrome (199).

#### 4.5.3 Oridonin

Oridonin, a diterpenoid from Rabdosia rubescens, selectively inhibits NLRP3 by covalently binding cysteine 279 in the NACHT domain, thereby disrupting the NEK7–NLRP3 interaction (200). It does not affect AIM2 or NLRC4 inflammasomes. Oridonin (IC50 = 0.5 μM in BMDMs) reduces IL-1β secretion and neutrophil infiltration in MSU-induced peritonitis and improves insulin sensitivity in high-fat diet models (200).

#### 4.5.4 OLT1177

OLT1177 is an orally active β-sulfonyl nitrile compound that inhibits NLRP3 ATPase activity (IC50 = 1 nM in J774A.1 cells) (201). It reduces IL-1β and IL-18 release after both canonical and non-canonical activation without affecting NLRC4 or AIM2. OLT1177 is currently in phase II clinical trials (201).

#### 4.5.5 INF39

INF39 is an acrylate derivative that inhibits both canonical and non-canonical NLRP3 pathways (IC50 = 10 μM in THP-1 cells) by altering NLRP3 conformation and blocking ATPase activity (202). It has shown protective effects in a rat model of inflammatory bowel disease but also inhibits NF-κB signaling.

#### 4.5.6 Sulforaphane

Sulforaphane, a compound found in cruciferous vegetables, reduces IL-1β levels and NLRP3 activation in MSU-induced gout models, partly by lowering mitochondrial ROS and inducing autophagy via the AMPK-Ulk1 pathway (203, 204). However, it also affects AIM2 inflammasome activation and therefore lacks NLRP3 specificity (205).

#### 4.5.7 Entrectinib

Entrectinib, an FDA-approved ROS1 inhibitor for ROS1-positive NSCLC, directly targets arginine 121 of NEK7, blocking the NEK7–NLRP3 interaction (206). It has shown efficacy in mouse models of LPS-induced inflammation, peritonitis, and T2DM. Two additional NEK7 inhibitors, HT-6184 (phase I) and MRT-3483 (preclinical), are under development (207).

In addition to the compounds described above, several other molecules—including C77, tranilast, MNS, and BOT-4-One—have been reported to inhibit NLRP3 inflammasome activation. Small-molecule inhibitors that directly target NLRP3 offer advantages over biologics, including oral bioavailability and potentially greater specificity. Several next-generation NLRP3 inhibitors (e.g., ZYIL1, DFV890, selnoflast, VTX3232, VTX2735, NT-0796, and NT-0249) are currently in clinical development, although their structures remain undisclosed in some cases. HT-6184 modulates both NEK7 and NLRP3 functions. Continued efforts to improve potency, selectivity, and safety profiles are expected to yield clinically viable agents for NLRP3-driven inflammatory diseases.

### 4.6 Emerging therapeutic approaches linked to NLRP3 modulation

In addition to direct NLRP3 inhibitors, several newer therapeutic strategies have shown potential to modulate NLRP3 inflammasome activity through indirect or complementary mechanisms. These approaches may offer advantages in terms of safety, specificity, or clinical translatability.

SGLT2 inhibitors have emerged as a promising class with NLRP3-modulating effects. Agents such as empagliflozin have been shown to reduce mitochondrial reactive oxygen species and inhibit NLRP3 inflammasome activation in models of HFpEF, contributing to improved cardiac function. These anti-inflammatory effects appear to be partly independent of glucose lowering and may involve improvements in myocardial energy metabolism (126).

The field of immunometabolism has revealed important connections between cellular metabolic state and NLRP3 inflammasome activity. Metabolic reprogramming toward glycolysis promotes NLRP3 activation, whereas activation of energy-sensing pathways such as AMPK and SIRT1 tends to exert inhibitory effects. Targeting these metabolic checkpoints may provide alternative strategies to modulate NLRP3-driven inflammation without directly inhibiting the inflammasome complex itself (72).

Finally, increasing attention is being directed toward cell-specific inflammasome signaling. NLRP3 exerts divergent roles in different cardiac cell populations, including macrophages, cardiomyocytes, fibroblasts, and endothelial cells. Future therapeutic strategies may benefit from cell-targeted delivery systems or cell-specific genetic approaches to achieve greater precision while minimizing off-target effects on systemic immune function (8). These emerging approaches highlight the expanding therapeutic landscape beyond direct NLRP3 blockade and warrant further investigation in both preclinical and clinical settings.

A summary of the main NLRP3 inflammasome inhibitors, including their targets, experimental models, main effects, is presented in **Table 1**.

**Table 1:** Main NLRP3 inflammasome inhibitors in cardiovascular disease research.

Inhibitor	Primary target	Molecular features and properties	Main models	Main effects or pathways	Ref.
MCC950	NLRP3 NACHT domain	Diarylsulfonylurea small molecule; orally bioavailable in preclinical models; IC_50_ 7.5 nM (BMDM)/8.1 nM (human macrophage); Phase II clinical trial discontinued due to hepatotoxicity	BMDMs; human macrophages; ApoE^−^/^−^ mice (AS); TAC mice (HF); CAPS mouse model	MCC950 directly binds the Walker B motif of the NACHT domain, blocking ATP hydrolysis and preventing NLRP3 oligomerization. It inhibits both canonical and non-canonical NLRP3 activation without affecting AIM2, NLRC4, or NLRP1. In TAC mice, MCC950 treatment increased LVEF and LVFS, reduced LVEDD, and attenuated myocardial fibrosis. In CREM transgenic mice, it reduced the incidence of spontaneous and induced AF.	(118, 119, 197)
CY-09	NLRP3 NACHT domain	Analog of CFTR(inh)-172 without CFTR inhibitory activity; small molecule; moderate aqueous solubility	LPS-primed BMDMs; gout patient synovial fluid cells; CAPS, T2DM, ApoE^−^/^−^ mice	CY-09 binds specifically to the ATP-binding motif of the NACHT domain and inhibits its ATPase activity, thereby blocking NLRP3 assembly and downstream pro-caspase-1 activation and IL-1β secretion. It does not affect LPS-induced TNF-α production or basal NLRP3 and pro-IL-1β expression.	(199)
Oridonin	NLRP3 Cys279	Diterpenoid from Rabdosia rubescens; forms partial covalent bond with Cys279; IC_50_ ~0.5 μM (BMDM); poor water solubility	BMDMs; ApoE^−^/^−^ mice (AS); MSU peritonitis mice; gout/T2DM mouse models	Oridonin covalently modifies Cys279 in the NACHT domain, disrupting the NEK7–NLRP3 interaction and selectively inhibiting NLRP3 without affecting AIM2 or NLRC4. In vivo, it reduced IL-1β secretion and neutrophil infiltration in MSU-induced peritonitis. In ApoE^−^/^−^ mice, it attenuated atherosclerosis and activated Nrf2, reducing Nrf2 ubiquitination and degradation.	(76, 200)
OLT1177	NLRP3 ATPase domain	β-sulfonyl nitrile compound; orally active; IC_50_ ~1 nM (J774A.1)/~1 μM (human macrophage); good aqueous solubility and oral bioavailability; Phase II ongoing	Mouse macrophages; human macrophages; non-reperfused AMI mice; HFrEF patients (Phase 1B, 14 days)	OLT1177 inhibits NLRP3 ATPase activity and prevents the formation of NLRP3-ASC and NLRP3-caspase-1 complexes, reducing IL-1β and IL-18 release after both canonical and non-canonical activation. In a mouse model of non-reperfused anterior wall MI, it preserved β-adrenergic receptor reactivity and prevented LV systolic and diastolic dysfunction. In a Phase 1B trial in HFrEF patients, 14-day treatment improved LVEF and exercise time with good tolerability.	(120, 121, 201)
INF150	NLRP3 NACHT domain	Small molecule; cell-permeable in macrophages; poor cardiomyocyte uptake	BMDMs; ex vivo isolated hearts	INF150 selectively inhibits NLRP3 in macrophages and confers protection in that cellular context, but fails to reduce infarct size in whole-heart ex vivo models owing to insufficient cardiomyocyte penetration. Its structural precursor INF195 retains cardioprotective activity.	(208)
INF195	NLRP3 (NACHT domain)	Small molecule; high aqueous solubility; good oral bioavailability; cardioprotective at 5–20 μM	THP-1 macrophages (in vitro); isolated mouse hearts (ex vivo: 30 min ischemia/1 h reperfusion)	INF195 inhibits NLRP3 and reduces myocardial infarct size in ex vivo I/R models. It decreased caspase-1 and IL-1β concentrations in cardiac tissue homogenates and showed cardioprotective effects even at the lowest tested dose of 5 μM.	(209)
INF39	NLRP3 ATPase domain	Acrylamide derivative; non-toxic; irreversible inhibitor; IC_50_ ~10 μM (THP-1); also partially inhibits NF-κB	THP-1 macrophages; DNBS-induced colitis rat model	INF39 irreversibly modifies NLRP3 conformation, inhibiting ATPase activity and reducing IL-1β release. It suppresses both canonical and non-canonical NLRP3 signaling in macrophages. Oral administration alleviated DNBS-induced colitis in rats. It should be noted that INF39 also partially inhibits the NF-κB pathway, limiting its selectivity for NLRP3.	(202)
Entrectinib	NEK7 Arg121	Small-molecule TKI; FDA-approved for ROS1-positive NSCLC; crosses blood-brain barrier; long half-life; repurposed use	BMDMs; LPS-induced systemic inflammation mice; peritonitis mice; T2DM mice	Entrectinib was found to directly target Arg121 of NEK7, blocking the NEK7–NLRP3 interaction and preventing subsequent inflammasome assembly and activation. In multiple mouse models including LPS-induced systemic inflammation, peritonitis, and T2DM, it showed significant anti-inflammatory effects, identifying NEK7 as a novel therapeutic target for NLRP3-associated diseases.	(206)
HT-6184	NEK7	Small molecule; good oral bioavailability; Phase I ongoing (NCT05447546); modulates both NEK7 and NLRP3 functions	Human THP-1 cells; mouse peritonitis model	HT-6184 inhibits the NEK7–NLRP3 interaction and blocks NLRP3 inflammasome assembly. Anti-inflammatory activity has been demonstrated in THP-1 cells and a mouse peritonitis model. A Phase I clinical trial is ongoing to evaluate safety and tolerability.	(207)
JC124	ASC	Small molecule; IC_50_ ~3.25 μM (mouse macrophages); cell-permeable; does not affect NLRP3 ATPase activity	Mouse macrophages (constitutively active NLRP3); TBI mouse model	JC124 directly binds the NLRP3 inflammasome complex and inhibits ASC speck formation and oligomerization without affecting NLRP3 ATPase activity. In a mouse model of TBI, it significantly reduced the expression of NLRP3, ASC, IL-1β, TNF-α, iNOS, and caspase-1.	(192)
VX-765	Caspase-1	Peptidic prodrug; converted to active metabolite by plasma esterases; moderate oral bioavailability; clinical development halted due to toxicity	BMDMs; ApoE^−^/^−^ mice (AS); rat I/R model (combined with P2Y12 antagonist); in vitro VSMC pyroptosis model	VX-765 inhibits caspase-1 and thereby blocks the maturation and secretion of IL-1β and IL-18. It reduced ox-LDL-induced VSMC pyroptosis in vitro and attenuated atherosclerosis in ApoE^−^/^−^ mice independently of lipid-lowering. When combined with a P2Y12 receptor antagonist in a rat I/R model, it further reduced infarct size and preserved cardiac function.	(95, 129, 196)
BAY 11-7082	IκB kinase (IKK)/NLRP3 ATPase domain	Small molecule; IKK inhibitor; also alkylates cysteine residues in the NLRP3 ATPase domain; moderate solubility	BMDMs; MI rat model (pretreatment 30 min before infarction); EAE and diabetic nephropathy models	BAY 11–7082 inhibits IκBα phosphorylation, thereby suppressing NF-κB-mediated priming of NLRP3. It additionally alkylates cysteine residues in the NLRP3 ATPase domain, impairing its ATPase activity. In a rat MI model, pretreatment with BAY 11–7082 significantly reduced infarct size and preserved cardiac function.	(95, 185, 186)
Colchicine	Tubulin/ASC	Alkaloid; moderate aqueous solubility; good oral absorption; FDA-approved (gout, recurrent pericarditis); non-specific anti-inflammatory	AMI mice (0.1 mg/kg/d × 7 d); TAC mice; HFpEF rats; STEMI patients (Phase II RCT, n=151); LoDoCo trial; post-ablation AF RCTs	Colchicine inhibits microtubule polymerization, disrupting ASC speck formation, NLRP3 inflammasome assembly, and mitochondrial transport. It also inhibits P2X7R activation and PYD–PYD interactions. In AMI mice, it reduced NLRP3 activation and infarct size. A Phase II trial in STEMI patients showed reduced infarct size and inflammatory biomarkers. The LoDoCo trial demonstrated reduced cardiovascular rehospitalizations. Clinical trials have also evaluated its use for prevention of AF recurrence after pulmonary vein isolation.	(96–98, 122, 123, 150–152)

Tip: AMI, acute myocardial infarction; AS, atherosclerosis; AF, atrial fibrillation; BMDM, bone marrow-derived macrophage; CAPS, cryopyrin-associated periodic syndrome; CKD, chronic kidney disease; EAE, experimental autoimmune encephalomyelitis; HF, heart failure; HFrEF/HFpEF, HF with reduced/preserved ejection fraction; I/R, ischemia-reperfusion; LV, left ventricular; LVEF, LV ejection fraction; MI, myocardial infarction; MSU, monosodium urate; NASH, non-alcoholic steatohepatitis; NSCLC, non-small cell lung cancer; STEMI, ST-elevation MI; T1/2DM, type 1/2 diabetes mellitus; TAC, transverse aortic constriction; TBI, traumatic brain injury; TKI, tyrosine kinase inhibitor; VSMC, vascular smooth muscle cell. 5 Challenges and future directions.

Extensive research over the past two decades has established the NLRP3 inflammasome as a key contributor to sterile inflammation in CVDs, from early atherosclerosis to HF, AF, and DCM. As summarized in this review, NLRP3 activation drives endothelial dysfunction, vascular smooth muscle cell phenotypic switching, foam cell formation, myocardial pyroptosis, adverse remodeling, and atrial electrical remodeling through the release of IL-1β and IL-18 and gasdermin D (GSDMD)-mediated pyroptosis. The CANTOS trial provided the first clinical evidence that targeting IL-1β downstream of NLRP3 reduces major adverse cardiovascular events in patients with atherosclerotic CVD. Despite these advances, several important challenges must be overcome before NLRP3-targeted therapies can be widely adopted in clinical practice. This final section outlines the main bottlenecks and proposes directions for future research to facilitate clinical translation.

### 5.1 Core challenges

#### 5.1.1 Mechanistic heterogeneity across CVD subtypes

NLRP3 inflammasome activation and regulation vary considerably across different CVD subtypes, cell types, and disease stages. In atherosclerosis, activation is largely driven by cholesterol crystal-induced lysosomal damage; in myocardial infarction and ischemia-reperfusion injury, it is dominated by mitochondrial dysfunction and ROS; in HF, it is sustained by chronic mitochondrial stress and TGF-β/Smad signaling; and in AF, it is linked to calcium dysregulation and electrical remodeling. However, the cell-type-specific roles of NLRP3 in macrophages, endothelial cells, cardiomyocytes, and fibroblasts remain incompletely defined. The contribution of non-canonical pathways (e.g., caspase-11/4/5) and the crosstalk between NLRP3 and other pathways (e.g., metabolic signaling, ferroptosis, and autophagy) are also poorly understood. These gaps hinder the development of precise, disease-specific interventions.

#### 5.1.2 Gaps between preclinical models and human disease

Most mechanistic and therapeutic studies rely on inbred mouse models (e.g., ApoE-/- for atherosclerosis, transverse aortic constriction for HF, coronary ligation for MI), which only partially recapitulate human CVD pathophysiology. Mouse atherosclerotic plaques lack the complex necrotic cores and fibrous caps typical of human lesions, and immune cell composition and inflammatory kinetics differ between species. In addition, most preclinical studies use prophylactic treatment, whereas clinical therapy is usually initiated after disease is established. These discrepancies likely contribute to the failure of many promising compounds to show similar efficacy in human trials. Long-term safety data for NLRP3 inhibitors in chronic CVDs are also limited.

#### 5.1.3 Challenges in therapeutic specificity and safety

Developing NLRP3-targeted drugs faces major hurdles in specificity, safety, and precision. Many small-molecule inhibitors lack absolute selectivity and exhibit off-target effects. Even MCC950, despite high preclinical selectivity, was discontinued in phase II trials due to hepatotoxicity. Systemic blockade of NLRP3 or IL-1β increases the risk of serious infections, as observed in the CANTOS trial. Furthermore, optimal dosing regimens differ markedly between acute conditions (e.g., short-term high-dose intervention in MI) and chronic diseases (e.g., long-term low-dose maintenance in HF), but evidence-based, subtype-specific protocols have not been established. The therapeutic potential of NLRP3 inhibitors in HF with preserved ejection fraction (HFpEF) and AF also remains largely untested in large trials.

#### 5.1.4 Limitations in biomarkers and clinical trial design

The absence of NLRP3-specific biomarkers hinders precision medicine. Most trials rely on high-sensitivity C-reactive protein (hs-CRP) as a surrogate marker, which is not specific to NLRP3 activation. This limits patient stratification and real-time monitoring of treatment effects. In addition, existing clinical studies are often small, single-center, short-term phase I/II trials. Few large-scale, multicenter phase III randomized controlled trials with hard endpoints (e.g., major adverse cardiovascular events, mortality, HF hospitalization) have been conducted, and most lack stratified analyses based on age, sex, comorbidities, or genetic background.

### 5.2 Future directions

#### 5.2.1 Mapping spatiotemporal and cell-specific networks

Advances in single-cell RNA sequencing, spatial transcriptomics, spatial proteomics, and live-cell imaging offer powerful tools to resolve the heterogeneity of NLRP3 activation. Future studies should map the spatiotemporal dynamics of NLRP3 across cell types, disease stages, and tissue microenvironments in both human CVDs and preclinical models. Systematic investigation of crosstalk between NLRP3 and metabolic pathways, other forms of cell death, and adaptive immunity will help identify more specific downstream targets. Epigenetic and non-coding RNA regulation of NLRP3 in CVDs also warrants further exploration.

#### 5.2.2 Developing specific biomarkers for patient stratification

Reliable NLRP3-specific biomarkers are urgently needed. Promising candidates include circulating ASC specks, N-terminal GSDMD fragments, mature IL-1β/IL-18, cell-type-specific methylation signatures, and NLRP3-related molecules in plasma exosomes from endothelial cells or cardiomyocytes. Integrating these biomarkers with multi-omics data will enable precise patient stratification, identify individuals most likely to benefit from NLRP3-targeted therapy, and allow dynamic monitoring of treatment response.

#### 5.2.3 Advancing next-generation therapeutics

To improve specificity and safety, future efforts should focus on:

1. Highly selective allosteric NLRP3 inhibitors, particularly those targeting the NLRP3–NEK7 interaction;
2. Tissue- or cell-specific delivery systems (e.g., nanoparticles, antibody-drug conjugates, or AAV-mediated approaches) to achieve local inhibition while minimizing systemic exposure;
3. Drug repurposing and combination therapy, evaluating existing agents (e.g., colchicine, SGLT2 inhibitors, metformin) for NLRP3-related benefits and testing rational combinations with lipid-lowering or antiplatelet therapies; and
4. Optimization of natural compounds with NLRP3 inhibitory activity through target identification, structural modification, and rigorous clinical validation.

#### 5.2.4 Improving clinical trial design

Future trials should adopt biomarker-driven designs to enrich for responsive patients, conduct large-scale, long-term phase III studies with hard clinical endpoints in unmet areas (e.g., HFpEF, post-MI remodeling, post-ablation AF), and develop stage- and subtype-specific dosing regimens. Dedicated studies in special populations (elderly patients, those with diabetes or chronic kidney disease) are also needed to generate evidence for personalized treatment.

#### 5.2.5 Broadening therapeutic applications

Beyond established indications, NLRP3 inhibitors may have value in primary prevention in high-risk populations, perioperative myocardial protection, reduction of in-stent restenosis or post-procedural AF, and treatment of rare inflammatory CVDs such as recurrent pericarditis and cardiac sarcoidosis.

### 5.3 Summary and prospects

The NLRP3 inflammasome has emerged as an important therapeutic target in cardiovascular diseases, with the CANTOS trial providing the first clinical proof-of-concept that targeting inflammation downstream of NLRP3 can improve cardiovascular outcomes. However, several critical translational barriers must be overcome before NLRP3-targeted therapies can be widely adopted in clinical practice. These include the lack of highly selective and safe small-molecule inhibitors, the risk of impairing host defense with systemic IL-1β or NLRP3 blockade, difficulties in achieving tissue- or cell-specific targeting, and the limited predictive value of current preclinical models for human disease.

Looking ahead, future research should prioritize several key areas. First, the development of next-generation inhibitors with improved selectivity and tissue-targeted delivery systems is essential to enhance the therapeutic index. Second, the identification and validation of NLRP3-specific biomarkers will be critical for patient stratification and real-time monitoring of treatment response. Third, well-designed, biomarker-driven phase III clinical trials focusing on unmet needs—such as heart failure with preserved ejection fraction and post-myocardial infarction remodeling—are urgently needed. Finally, integrating multi-omics technologies with mechanistic studies will help clarify cell-specific and disease subtype-specific roles of the NLRP3 inflammasome, thereby guiding more precise therapeutic strategies.

Addressing these challenges through coordinated basic, translational, and clinical efforts will be necessary to fully realize the potential of NLRP3-targeted therapy in cardiovascular medicine.
